# Student challenges and learning strategies at Hong Kong EMI universities

**DOI:** 10.1371/journal.pone.0251564

**Published:** 2021-05-07

**Authors:** Jack Pun, Xina Jin

**Affiliations:** Department of English, City University of Hong Kong, Hong Kong SAR, China; Lingnan University, HONG KONG

## Abstract

The rapid trend towards globalization has led to the expansion of English as Medium of Instruction (EMI) in tertiary education. The academic challenges faced by non-native speakers have been broadly discussed in Anglophone countries, whilst those learning through EMI in the Chinese context are still underexplored. To examine Chinese EMI university students’ perceived language challenges in learning, as well as their language-related learning strategies, this study investigated 73 students studying at EMI universities in Hong Kong, taking account of their gender, EMI experience in higher education, and English exposure prior to tertiary education. Participants completed a survey to provide self-evaluations of their academic situation and their perceptions of their disciplinary learning. The study found that students perceived a relatively low level of language and learning challenges, and they showed a preference for using their second language (L2)-related learning strategies over strategies related to their first language. Specifically, male students tended to be more actively engaged in communication with their peers than females, and were more likely to search for additional learning support in English. In addition, first-year undergraduates perceived a greater degree of challenges in knowledge application and relied more on L2-related learning strategies than their senior counterparts. Although the importance of English exposure prior to higher education has been highlighted in many existing studies, this study found that prior exposure to English was neither connected with students’ perceived challenges nor their learning strategies.

## Introduction

The rapid trend towards globalization has led to an educational transformation in medium of instruction, and enabled people all over the world to share ideas and information with a common language. This phenomenon contributes to the expansion of English as Medium of Instruction (EMI), which allows for a more widespread adoption of English when teaching content knowledge in nations or jurisdictions where the dominant official language is not English [1–3]. To date, there has been no restriction on the establishment of EMI; generally, integrated EMI education is widely applied at the tertiary level around the world [1]. University students are expected to benefit from EMI programmes in terms of content learning as well as additional opportunities to learn English in their discipline [4]. From a psychological perspective, students’ eagerness for a promising future may stimulate their motivation to learn English through an additional language acquisition, which is important for learning subjects that matter in the EMI context [5–7].

Stakeholders engaging with the education sector generally reflect supportive attitudes towards EMI instruction, as it could bring potential benefits not only to students but also to educational institutions [8–11]. For example, students’ linguistic and professional skills, which are necessary for their future employment, can be improved through massive exposure to L2 [9]. For university administrators, EMI programmes provide an immediate path to gaining rapid status in global rankings so that they can attract more foreign elites as faculty members and international students [12]. However, such a win-win scenario is only likely to happen in the context of the perfect readiness each participant, and the ambivalence of successful EMI implementation is a theme that has been continuously discussed in recent studies [10]. Many studies have emphasized that the success of EMI programmes can be achieved on the basis of certain conditions: first, students’ English proficiency must have reached a certain threshold for their comprehension of content instruction in English [13–15]; second, EMI lecturers must be equipped with fluent English communication skills, as well as professional knowledge in their disciplines and the pedagogical skills to provide effective scaffolding for both the content and language [16,17]; and third, only when universities provide sufficient teaching resources can EMI lecturers receive the necessary professional development to meet students’ learning needs [18,19]. For those whose first language is not English, this set of conditions might be harder to be fulfilled; the adoption of EMI education has therefore never been as smooth as expected. Many studies have identified the potential problems caused by language transitions from their first language (L1) to English [4,20]. By investigating students’ academic challenges, as well as the strategic tactics used by students for learning, this study aims to develop a better understanding of the effectiveness of EMI in higher education in Hong Kong.

### The relation between EMI experiences and English abilities

The importance of EMI students’ English abilities has primarily been highlighted in terms of their academic learning [17,18,21]. For example, Kirkgöz found that the majority of Turkish university students responded to the gap between their current English level and the language requirements of their EMI courses, such as the English used in their disciplines [22]. In a similar vein, Byun et al. indicated that English language support was needed for university students who did not achieve the threshold level of English competency, in order to fulfil their academic tasks in an English-only environment [23]. University students may otherwise encounter language challenges if there is no suitable language support in place to develop English language skills required in their discipline [19,20,24].

With the acknowledgement of the impact of English as an L2 in content learning, a growing number of researchers in the past few years have urged more attention to be given to the linguistic and learning challenges imposed by the transition in medium of instruction [4,19,25–27]. The findings of these existing studies suggest that university students with limited competence in L2 usually encounter more language problems than their counterparts in classroom interactions, group work, content understanding, reading learning materials and writing assignments. Moreover, there is consistent evidence that multidimensional factors somewhat relate to learners’ English skills. For instance, Lin and Morrison indicated that massive exposure to English prior to university was essential to the transition into EMI tertiary education [28]. In their research, university students with Chinese-medium secondary school backgrounds were found to demonstrate a lower proportion of ‘satisfactory’ scores in receptive academic vocabulary than those graduating from EMI secondary schools. In their actual academic performance, the latter group of students outperformed significantly on the content-subject exams. Hence, without extensive exposure to English, university students tended to experience greater difficulties in coping with productive tasks such as academic writing.

### Noteworthy factors that affect EMI students: Gender and discipline

Individual characteristics such as gender have been revealed as significant parameters related to university students’ motivation with regard to language learning, even when considered apart from the sociological perspective [29,30]. Gu concluded that amongst Chinese tertiary students who speak English as a foreign language, female learners performed better in English proficiency tests than their male counterparts [31]. Meanwhile, Lasagabaster stated that gender differences in L2 learning motivation and attitudes did not exist in the university contexts [32]. Thus, students’ mentality in using L2 for education did not appear to account for such differentiation, and the lack of consensus in previous findings draws attention to researching the impact of gender on EMI education. Moreover, research on EMI education has rarely focused on gender effects in learning, and little evidence can be empirically obtained for further details. The latest research conducted by Macaro and Akincioglu included gender as one of the variables for exploring students’ perceptions of EMI programmes at Turkish universities [1]. Female students were found to possess a stronger belief that EMI as a motivating factor would bring positive benefits in language acquisition. However, in terms of the actual perceived difficulties, there were no significant differences between males and females, implying that students’ thoughts on EMI advantages do not change their self-perceptions on the difficulties encountered in EMI learning.

Just as importantly, students’ degree of perceived challenges differs according to the discipline that matters to their learning [33]. For example, science is an academically demanding subject that not only requires students to develop conceptual understandings but also involves learning processes in explaining complicated phenomena. The linguistic challenges science students of whom are learners of English as a second language (ESL) were well illustrated in previous research: scientific writings are *“difficult to read … it is important to stress that it is not only ESL students who find problems with scientific English—so also do many for whom English is the mother tongue”* [34]. Therefore, it is necessary to inclusively account for the elements mentioned above in order to examine the specific language challenges faced by students studying in a particular social and cultural background.

### L1 support as a complementary learning strategy

In the EMI context, it is believed that students’ academic benefits can accrue from the massive English exposure. The reason why for students struggle in English-only classrooms is mainly due to their lack of English ability. It is therefore reasonable to adopt assistance from L1 as an additional instrument for content comprehension. A number of studies have highlighted the importance of L1 adoption in the initial stage of EMI education, and some consider L1 to be strategically indispensable in the EMI learning environment [35–40]. Despite the significance of L1 support in EMI, an L2-only academic setting remains to be favoured by most stakeholders, such as teachers and students [29,41]. For example, evidence from research shows that L1 information on L2 lexes could benefit vocabulary retention [42–45] and promote L2 production [46]. Lin and Morrison [28] has confirmed the positive connection between L1 use and L2 development in Hong Kong EMI science classrooms. In light of their research findings, L1 intervention could help science students with poor English proficiency to access more technical terms in English and understand the language style used in scientific discourse in terms of content comprehension [28]. For those who are deficient in English, L1 adoption could also provide supplementary explanations of academic knowledge. Namely, students can draw from their familiar native language to explain, translate, and elaborate the L2-mediated knowledge taught in the classroom [47].

In a similar vein, Artieda’s findings also suggest that L1 literacy levels are fundamental for academically disadvantaged learners who benefit little from education in L2, yet students who have already gained intermediate L2 proficiency might have overcome the gap in making progress in a second language [48]. Scott and De La Fuente examined how college students used L1 and L2 to solve grammar problems [49]. Their results revealed that reading, thinking and talking appeared in an integrated manner among students who were allowed to use L1, whereas these processes were sequential in their counterparts, indicating that using L1 might help to reduce cognitive overload in L2 tasks. Kim, Kweon and Kim conduct a survey at three engineering universities to explore students’ perceptions of L1 use in EMI programmes [50]. Nine out of ten students were enthusiastic about the use of L1 over only English-mediated classes. They believed that L1 could facilitate content learning, and the strategy of L1 intervention supported the explanation for difficult content and materials. However, the aforementioned research was mostly conducted via a qualitative approach. To generalize our understanding of the positive effect of L1 on students’ academic studies, it is necessary to examine whether EMI students truly prefer to involve L1 in their learning.

### Research questions

English has long been a lingua franca in Hong Kong due to its colonial history. Although more than 80% of local people speak Cantonese for daily communication, English is one of the official languages that is widely utilized in many social activities. Students in Hong Kong have frequently been exposed to EMI before entering higher education, yet their levels of English proficiency cannot be guaranteed to be equal due to the different language policies established among the local secondary schools [18,51]. Hong Kong’s EMI policies greatly contributed to the unbalanced academic achievements in local universities, where almost all of the courses are provided in English only. Ironically, there is a large gap of research on EMI education in tertiary level since extant studies on Hong Kong EMI education have mostly been conducted at the secondary level [52–54]. To elicit evidence-based information on Hong Kong university students’ academic learning, this study set out to examine Hong Kong EMI university students’ challenges in terms of language and study, as well as their tendency to use language-related strategies to aid their studies, with the factors of gender and EMI experience (before and after university) taken into account. Thus, this research aims to answer the following research questions:

1. How do university students’ perceived language and learning challenges differ by gender, EMI experience in higher education and English exposure prior to university? And what is the extent of these perceived challenges?
2. How do university students’ learning strategies differ by gender, EMI experience in higher education and English exposure prior to university?

## Method

This study has been approved by the authors’ institutional review board—College Human Subjects Ethics Sub-Committee, College of Liberal Arts and Social Sciences at the City University of Hong Kong.

### Participants

As mentioned above, academically demanding disciplines may lead to more challenges in the EMI context. Content learning subjects, for example, science is especially complicated due to the abstract concepts and technical terminologies. ESL science students are often criticised for their low competence in scientific writing. Therefore, for this study, the researchers decided to focus on the population of science students for investigation. This study was conducted via a quantitative research method. Seventy-three students who were studying in science disciplines (e.g., engineering or natural sciences) were randomly recruited for the survey from two local, publicly-funded EMI universities. Of the participants, 23 (31.5%) are female, 50 (68.5%) are males. 51 (69.9%) were first-year junior undergraduates while the rest (n = 22, 30.1%) are non-freshman. Most of the students (n = 69, 93%) indicated that Cantonese is their first language, the rest of students (n = 4, 7%) speak Mandarin as their first language.

### Instruments

The questionnaire survey was adapted from existing scales, thus the validity and reliability of which have been statistically confirmed in previous studies in students’ perceptions and strategies of EMI learning [55–57]. It covers 149 items in total, with 7 questions related to demographic information, 142 items related to self-assessments of one’s situation (e.g., experience with EMI education prior to university, the degree of English usage in current science classrooms, English use inside and outside of university, and the medium of instruction interactions in the science classroom) and perceptions of major content-subject learning (e.g., degree of English difficulties in speaking/writing activities, English challenges in learning science, the importance of English in the study of science, the frequency and usefulness of strategy use related to the study of science, etc.). The questionnaire is submitted as a supporting document with the items that are highlighted in the analysis. Content validity was repeatedly checked by an expert panel consisting of science lecturers and education researchers. Pilot-tests were conducted on 48 volunteer undergraduates to ensure the feasibility of comprehending and completing the survey.

As shown in Table 1, the selected four scales fell into three categories: language challenges (speaking and writing), learning challenges and learning strategies. The first scale measures students’ difficulties in using spoken English, which consists of 7 items divided into two subscales. The participants were asked to indicate on a 5-point Likert scale of the extent to which they experienced difficulties in each case (e.g., presenting ideas/arguments/information to peers or presenting information to scientists/technicians), with a higher score indicating greater difficulty. The second scale was used to infer students’ degree of difficulty in logical writing (8 items) with the same rating scale (e.g., organizing ideas clearly and logically, summarizing ideas from sources, linking ideas from different sources, referring to sources correctly, and using an appropriate academic style). The third scale consisted of 12 statements that fall into three subscales measured with another 5-point Likert scale, which was used to rate the extent to which students faced challenges while learning scientific content knowledge (e.g., challenges in understanding course content and discipline-specific facts, challenges in calculating and solving problems in English, and challenges due to the teaching methods/style of an individual science teacher). The last scale adopted in this research aimed to elicit the participants’ tendency to choose language-related strategies to facilitate their study of science, which consisted of 6 items (e.g., requesting additional English explanations from science teachers, requesting additional Chinese explanations from science teachers, requesting lesson materials printed in bilingual languages, and asking science teachers for feedback and writing corrections in English). Two subscales were formed, and the participants are required to indicate the extent to which they use the learning strategies presented in each statement on a 5-point Likert scale, with a higher score indicating more frequent selection.

**Table 1:** The scale components, survey items and their internal consistency.

Scale (Number of items)	Cronbach’s α	Sample of item
**Language challenges**	**.84**	
Communication with peers (5)	.82	Presenting information to peers/classmates.
Communication with professionals (2)	.80	Communicating with scientists.
Logical writing (8)	.89	Organizing ideas clearly & logically.
**Learning challenges**	**.86**	
Content comprehension (4)	.77	Understanding course content and discipline-specific facts.
Knowledge application (5)	.83	Calculating and solving problems in English.
Learning adaptability (3)	.72	Learning from the teaching methods/style of an individual teacher.
**Learning strategies**	**.79**	
Searching for additional L2 support (4)	.72	Requesting additional English explanations from the science teacher
Searching for additional L1 support (2)	.75	Requesting lesson materials printed in bilingual languages (e.g., L1 and L2)

### Data collection and analysis

All the questionnaires were paper-and-pencil based, disseminated and completed during a scheduled appointment. There was no time limit set by the researchers, and the survey questionnaires were collected directly by the researchers upon completion by the respondents.

Students in this study were grouped according to three characteristics: gender, EMI experience in higher education, and English exposure prior to university education. The collected data were analysed using SPSS 21.0. First, the data were computed for each subscale score by averaging the participants’ ratings of the corresponding items. Regarding academic year, the coding was based on students’ indicated years of experiences; students who indicated in their first year of learning in the self-reported questionnaire were coded as freshman, and the others were coded as non-freshman. Students were divided into two categories according to the average value of the items concerning their language use from kindergarten to senior secondary level: low-exposure students and high-exposure students. Then, to explore whether participants of different genders (i.e., male vs. female), with different EMI experiences in higher education (i.e., freshman vs. non-freshman), and with different levels of English exposure prior to university (i.e., low exposure vs. high exposure) differ in their experience of challenges in language and learning, as well as their language-related learning strategies, a series of independent *t*-tests were performed to compare the means of each pair of groups. We set α (2-tailed) as 0.05 for all statistical tests.

## Results

### Language challenges perceived by the students

Table 2 shows the descriptive statistics of the *t*-test results for science students’ perceptions of their language challenges. There was a statistically significant difference between female and male students in terms of communication with peers, t(71) = 2.89, *p* = 0.005, d = 0.72. Cohen’s d is the appropriate measure for *effect size* when comparing the standardized difference between two means of groups [58]. It is generally accepted that d = 0.2 represents a small effect, whereas d = 0.5 and d = 0.8 are considered medium and large effects, respectively. Thus, the results show that the effect of gender on the outcome is quite large. Regardless of the relatively lower level of challenges in speaking and writing perceived by both males and females, the mean scores for males implies a better capability to handle English-related tasks in comparison to females.

**Table 2:** Descriptive statistics and *t*-test results for language challenges (female vs. male).

scales	Female		Male		t	df
	M	SD	M	SD		
Communication with peers	3.14	0.51	2.73	0.59	2.89**	71
Communication with professionals	2.89	0.52	2.86	0.73	0.16	69
Logical writing	2.98	0.46	2.89	0.74	0.55	70

1 = very easy, 2 = easy, 3 = neutral, 4 = difficult, 5 = very difficult;

* *p*<0.05;

** *p*<0.01;

*** *p*<0.001.

Table 3 presents descriptive results for the differences in perceived language challenges between freshmen and non-freshmen. No statistically significant difference was found among the subscales. In total, both groups of students share similar scores in all three subscales, but the mean score for non-freshmen indicates a slightly lower degree of difficulty communicating with professionals than freshmen experience.

**Table 3:** Descriptive statistics and *t*-test results for language challenges (freshmen vs. non-freshmen).

Scales	Freshman		Non-freshman		t	df
	M	SD	M	SD		
Communication with peers	2.85	0.62	2.87	0.54	-0.15	71
Communication with professionals	2.92	0.71	2.76	0.56	0.91	69
Logical writing	2.90	0.61	2.95	0.78	-0.34	70

1 = very easy, 2 = easy, 3 = neutral, 4 = difficult, 5 = very difficult; * *p*<0.05; ** *p*<0.01; *** *p*<0.001.

In terms of science students’ English exposure prior to studying at university, as shown in Table 4, the descriptive statistics reveal higher mean scores for the low-exposure group on all three subscales than for the high-exposure group, which means that students who were relatively less exposed to English-medium instruction before studying at tertiary level might encounter more complicated problems in language-related practices than their counterparts. However, the differences in the mean scores are not remarkable and are not statistically significant.

**Table 4:** Descriptive statistics and *t*-test results for language challenges (low exposure vs. high exposure).

Scales	Low exposure		High exposure		t	df
	M	SD	M	SD		
Communication with peers	2.92	0.52	2.77	0.69	1.08	71
Communication with professionals	2.96	0.68	2.74	0.64	1.39	69
Logical writing	2.95	0.65	2.87	0.69	0.51	70

1 = very easy, 2 = easy, 3 = neutral, 4 = difficult, 5 = very difficult; * *p*<0.05; ** *p*<0.01; *** *p*<0.001.

### Learning challenges perceived by the students

Regarding students’ perceived challenges in learning science, Table 5 summarizes the descriptive statistics and *t*-test results for the comparison between female and male students. As revealed by the mean scores, the female group had comparatively better ability to comprehend content as well as adapting their learning to the dynamic academic environment, yet this advantage is not visible in the knowledge application subscale. The *t*-test results between the two groups revealed no statistically significant differences.

**Table 5:** Descriptive statistics and *t*-test results for learning challenges (female vs. male).

Scales	Female		Male		t	df
	M	SD	M	SD		
Content comprehension	2.72	0.70	2.74	0.59	-0.17	69
Knowledge application	2.83	0.80	2.80	0.62	0.13	70
Learning adaptability	2.74	0.85	2.92	0.68	-0.96	70

1 = not challenging, 2 = slightly challenging, 3 = challenging, 4 = very challenging, 5 = most challenging.

* *p*<0.05; ** *p*<0.01; *** *p*<0.001.

Table 6 presents the descriptive statistics for the same subscales with regard to learning challenges perceived by freshmen and non-freshmen. There is a statistically significant difference between the two groups in knowledge application, t(70) = 2.89, *p* = 0.05, d = 0.50, a moderate effect size, indicating that freshmen need more time and space to become comfortable addressing learning issues in English than non-freshmen. A similar trend was also found in content comprehension but with minimal inter-group differences. However, the mean score for learning adaptability is greater in the freshman group, which means that freshmen may in fact encounter fewer difficulties in adapting to the EMI context.

**Table 6:** Descriptive statistics and *t*-test results for learning challenges (freshman vs. non-freshman).

Scales	Freshman		Non-freshman		t	df
	M	SD	M	SD		
Content comprehension	2.75	0.63	2.71	0.62	0.19	69
Knowledge application	2.91	0.69	2.58	0.59	2.08*	70
Learning adaptability	2.81	0.73	2.98	0.75	-0.94	70

1 = not challenging, 2 = slightly challenging, 3 = challenging, 4 = very challenging, 5 = most challenging.

*p<0.05;

** *p*<0.01;

*** *p*<0.001.

Table 7 shows the results of the *t*-tests comparisons based on science students’ English exposure. As shown in the data, the average gap between the two groups was not significant. The mean scores for science students with lower English exposure indicate minute deficiencies in content comprehension and learning adaptability rather than in knowledge application. The *t*-test results reveal no significant difference between the groups on any subscales of the learning challenges.

**Table 7:** Descriptive statistics and *t*-test results for learning challenges (low exposure vs. high exposure).

Scales	Low exposure		High exposure		t	df
	M	SD	M	SD		
Content comprehension	2.74	0.59	2.73	0.67	0.036	69
Knowledge application	2.79	0.52	2.84	0.86	-0.31	70
Learning adaptability	2.88	0.71	2.84	0.79	0.21	70

1 = not challenging, 2 = slightly challenging, 3 = challenging, 4 = very challenging, 5 = most challenging.

*p<0.05; ** *p*<0.01; *** *p*<0.001.

### Learning strategies

In terms of learning strategies, Table 8 illustrates the lower mean scores for females on both subscales than for males. A significant gap was found in searching for additional L2 support, which means that male students search more for additional explanations for science content in English than female students do, t(68) = -2.91, *p* = 0.005, d = -0.76. The value of Cohen’s d indicates a high impact.

**Table 8:** Descriptive statistics and *t*-test results for science learning strategies (female vs. male).

Scales	Female		Male		t	df
	M	SD	M	SD		
Searching for additional L2 support	2.29	0.69	2.86	0.78	-2.91**	68
Searching for additional L1 support	2.11	0.94	2.50	1.01	-1.52	68

1 = never, 2 = rarely, 3 = sometimes, 4 = usually, 5 = always;

*p<0.05;

** *p*<0.01;

*** *p*<0.001.

Table 9 presents the descriptive statistics and *t*-test results for the freshman and non-freshman groups. The results show a statistically significant difference between the two groups in searching for additional L2 support, which means that first-year science students devoted more effort to learning science in L2 in the EMI context than non-first-year students, t(68) = 2.11, *p* = 0.39, d = 0.54; the effect size is moderate. In addition, the data show that freshmen and non-freshmen sought L1-related assistance at similar frequencies.

**Table 9:** Descriptive statistics and *t*-test results for science learning strategies (freshmen vs. non-freshmen).

Scales	Freshman		Non-freshman		t	df
	M	SD	M	SD		
Searching for additional L2 support	2.82	0.81	2.40	0.68	2.11*	68
Searching for additional L1 support	2.38	1.00	2.39	1.01	-0.04	68

1 = never, 2 = rarely, 3 = sometimes, 4 = usually, 5 = always;

* *p*<0.05;

** *p*<0.01;

*** *p*<0.001.

As the descriptive statistics presented in Table 10 show, students with higher English exposure revealed higher mean scores on both subscales for learning strategies used in science courses. It seems that English exposure during compulsory education does not determine student preferences for language adoption in higher education at EMI universities. No statistically significant difference was identified from the *t*-test results between the groups.

**Table 10:** Descriptive statistics and *t*-test results for science learning strategies (low exposure vs. high exposure).

Scales	Low exposure		High exposure		t	df
	M	SD	M	SD		
Searching for additional L2 support	2.68	0.73	2.69	0.9	-0.05	68
Searching for additional L1 support	2.36	0.99	2.41	1.02	-0.19	68

1 = never, 2 = rarely, 3 = sometimes, 4 = usually, 5 = always; * *p*<0.05; ** *p*<0.01; *** *p*<0.001.

## Discussion

This study has yielded insights into science students’ learning situations at Hong Kong EMI universities based on the consideration of three main elements: gender, EMI experience in higher education, and English exposure prior to higher education. The initial descriptive statistics revealed that the mean scores for each student group were below 3, suggesting that learners perceived moderate challenges. In addition, students are inclined to look for additional L2 support while striving to do better in their academic practice. These findings are discussed in detail in terms of the two research questions: 1) students’ perceived challenges in language and learning; and 2) students’ learning strategies.

First, the overall level of language challenges indicated by the students was relatively low, suggesting that the majority of science students encountered little trouble in producing academic output using English. This finding is contrary to previous study by Evans and Morrision [21], which suggested that university students in Hong Kong experience quite a few language problems during their university studies, especially in their first-year learning period. In their research, secondary school background was an important factor that determined students’ mastery of vocabularies. Namely, students from English-medium schools had typically gained much more vocabularies than those from Chinese-medium schools. The lack of vocabulary knowledge, particularly in students who graduated from Chinese-medium schools, could in turn significantly impede their comprehension of lectures and learning materials, as well as their communications in the classroom. Surprisingly, in this study, neither students’ prior exposure to English nor EMI experience in university appears to be notably essential in science students’ language adaptation. Our findings may therefore suggest that idea expression through English is unlikely to be a significant burden in students’ academic life. Moreover, the results may infer that most science student participants in this study have surpassed the threshold for English proficiency required for EMI at the tertiary level and hence, they appeared to have skipped the transition point of language adjustment [59].

From the perspective of gender differences, both female and male students indicated experiencing difficulties to a similar extent in spoken and written English while conducting their academic activities. However, male students presented a better pattern than female students in peer communication, and the result is statistically significant. While the gender gap in second language acquisition has always been controversial, existing studies have provided a pivotal insight: we should refrain from biased views in terms of cognitive differences in gender [60]. Our finding differs from that of Arshad, Shahbaz and Al-Bashabsheh’s research, in which they claimed that females tend to be more stable in L2 writing [66]. Unlike the current study, Arshad et al.’s [61] study did not identify an explicit gap in L2 use between male and female learners. It is worth noting that communication in the ESL context contributes to the speakers’ willingness to talk to a certain extent [62]. With consideration of the linguistic, social and psychological variables, McIntyre et al. introduced several factors that may affect one’s willingness to communicate: the level of connection among the communications, the number of people involved in communication, the requirements of the environment, the skill of the speaker, and the communication topic [63]. In this research, the questionnaire items on communication challenges refer to the science classroom setting, which means males might not actually be better than females at using English, but females were likely to feel more stressed or shy when actively presenting their opinions in front of people. In this regard, Arshad et al. mentioned that male students express a stronger desire to speak and share information, and tend to react more confidently to social situations [49]. Therefore, it is necessary to encourage female students to actively share their opinions with their peers.

Second, this study found that the participants as a whole experienced few challenges in content learning. Among the participants, first-year undergraduates reported significantly greater challenges in terms of knowledge application than non-freshmen. As aforementioned, although participants have mostly attained an above-threshold level of English for exchanging ideas, as newcomers to the world of using only English as the medium of instruction, first-year students need a buffer period to adjust to the circumstances that require knowledge transformation to occur completely in L2. A number of studies conducted in English-speaking nations have identified negative experiences among first-year non-native students at universities [64,65]. These studies have consistently claimed that first-year undergraduates face more learning barriers than native counterparts in the English as a second/foreign language context [66]. According to Evans and Morrison, freshmen in Hong Kong EMI universities encounter many transitional challenges in understanding, comprehending, and adapting to new methods in learning [21]. However, these challenges could be overcome by hard work and the use of proactive learning strategies [18]. In terms of participants’ learning challenges, the findings of the present study support the assertion that most science students with EMI background are equipped with language ability to navigate their EMI transition in learning and eventually, benefit from EMI instruction through their efforts. In terms of gender, no differences in learning were identified, and this finding once again suggests that gender differences are not tenably related to cognitive issues in EMI tertiary education.

Lastly, a major finding regarding science students’ learning strategies showed that males tend to use L1 and L2 learning strategies more frequently, and a statistically significant gender difference was found in the frequency of L2-related learning strategies use in participants. In a similar vein, when asked about the frequency with which learning strategies were applied, freshmen revealed a statistically greater adoption of L2-related strategies than non-freshmen, which means that first-year students endeavoured to maintain English as the medium of knowledge acquisition. This finding is not consistent with those of previous studies conducted in the Hong Kong higher education setting [20,21]. For the majority of local students, English is rarely spoken outside of school premises or in their daily lives. It was reported that some local undergraduates were still accustomed to using materials written in Chinese, and listening and speaking in their L1. Meanwhile, previous literature has shown the significance of L1 use for ESL students, in which the adoption of L1 learning strategies potentially benefit their content comprehension [38,67–70]. An increased L1 usage can not only encourage students to achieve their own conceptual understandings through the use of higher-order pedagogical skills (clarification requests, confirmation checks and signals, and comprehension checks), but may also help integrate new knowledge into their existing knowledge in L1, thereby promoting a deeper understanding of science concepts and scientific process. While learning through a familiar language may be better for cognitive content mastery, students in this study were more prone to resolve learning issues using L2. For local students, a significant proportion of their academic and future working lives requires the use of English, such as in dealing with English meetings, documents, and emails. Such reality may have motivated science students who study at EMI universities in Hong Kong to place greater importance in L2 than L1 in their learning starting from their first year of studies. The motivation of learning in their L2 supports the institutional goal of nurturing global competitiveness, and facilitates the production of a massively demanded workforce in the scientific fields.

## Implications

A few implications can be obtained from the present research findings. Despite the conservative assertion of ESL science students may experience difficulties during their content learning, deploying full English immersion in higher education appears to be accepted and welcomed by globalized society, in which English exposure has been officially supported since early education. In a similar vein, our findings may infer that a majority of students who study at Hong Kong EMI universities are equipped with a threshold-level of L2 skills for content learning. However, since there exists a moderate gap between the perceived language and learning challenges between freshmen and non-freshmen, more instructional efforts should be made to resolve this problem. In addition, subject content is a major challenge student face during their learning process. Notwithstanding their prior exposure to English, the English level of ESL students may not be sufficient for them to master the specific terms and abstract concepts in different subjects. Using science subjects as examples, Halliday and Martin suggested that scientific language has a specific register, discourse and lexico-grammatical features [34]. The language demands required for understanding the abstract concepts and using specific terms in English classrooms are distinctive to the register used in daily English [34,71]. Therefore, teachers should incorporate more practical items to the English for Academic (EAP) courses and English for Specific purposes (ESP) courses based on ESL students’ actual needs. Lastly, due to the high demand of English level for teaching EMI subject-specific courses, specialized training on the strategies to cope with language-related challenges for both teachers and students is highly regarded [72,73]. By providing an evidence-based, pedagogically focused analysis of students’ perceptions, this research sheds light on ways to improve the quality of instructional practices in different EMI classrooms in Hong Kong and similar contexts around the world. Additionally, since our research mainly focuses on students’ English exposure, future studies could adjust different factors that are influential to not only students’ learning outcomes, but also their learning process.

## Conclusion

The current study conducted at EMI universities in Hong Kong aimed to investigate science students’ linguistic and cognitive challenges, as well as the learning strategies they adopted in an academic setting by comparing groups based on their gender, EMI experience in higher education, and English exposure prior to university study. This research found that participants overall perceived few challenges in EMI science classrooms, including in spoken and written English, and in content learning. In addition, students tended to use L2-related learning strategies more often than L1-related learning strategies. Group differences were identified according to gender and EMI experience. Specifically, males were more comfortable communicating with peers in the classroom, and tended to use L2 learning strategies more frequently than females. At the same time, first-year undergraduates perceived a greater degree of challenges in knowledge application, and they relied more on L2-related learning strategies than their non-first-year students. These findings provide new insights into science students’ levels of linguistic ability and academic adaptability in local EMI universities. However, the impact of these research findings should be interpreted with caution due to the methodological limitations. First, although the researchers were fully aware of the importance of sample size, the number of participants was not sufficient to provide strong evidence as a large sample could do. While the requirement of a minimum sample size for the t-test was fulfilled in each categorized group, the disparities of sample size in the two gender groups and two grade-level groups may have affected statistical accuracy of the results. This is also one of the reasons why the current study adopted several t-tests, rather than more advanced statistical analysis approaches. To adopt Multivariate analysis of variance (MANOVA), for example, the minimum sample size is required to exceed the current one. Hence, future studies could complement this limitation by increasing the sample size and conducting more sophisticated analysis. Second, the study was designed to reveal information about EMI science students. The current study, however, is not able to present broad explanations of or potential reasons for the findings. Therefore, further methodological approaches should be established to provide more detailed explanations. Qualitative research, such as follow-up interview studies, shall be considered in future research to address this limitation.

## Supporting information

S1 Questionnaire (DOCX)
