# Supplementary material for: Student challenges and learning strategies at Hong Kong EMI universities
Source: PLoS One. 2021 May 7;16(5):e0251564. doi: 10.1371/journal.pone.0251564 (PMC8104429; doi:10.1371/journal.pone.0251564)
Supplement: S1 Questionnaire — (DOCX) [file pone.0251564.s001.docx]

**Supporting Documents**

**Questionnaire**

*Include scales being used only

The information that you provide will be treated in the strictest confidence. Please answer each question by ticking the most appropriate box or filling in the blanks.

| 1. Gender: | ☐Female ☐Male | |
| --- | --- | --- |
| 1. Age: | ☐17 ☐18 ☐19 ☐20 ☐21 ☐22 ☐23 ☐24 ☐25 ☐26 Others:________ | |
| 1. Year: | ☐Y1 ☐Y2 ☐Y3 ☐Y4 ☐Y5 | |
| 1. College/School: |  | |
| 1. Department: | ☐ Architecture & Civil Engineering  ☐ Biomedical Engineering  ☐ Biomedical Sciences  ☐ Building Science & Technology  ☐ Chemistry  ☐ Computer Science  ☐ Electronic Engineering | ☐ Energy and Environment  ☐ Infectious Diseases & Public Health  ☐ Materials Science & Engineering  ☐ Mathematics  ☐ Mechanical Engineering  ☐ Physics  ☐ Systems Engineering & Engineering Management |
| 1. First language: | ☐Cantonese ☐Putonghua ☐English ☐Others:____________(please specify) | |
| 1. Place of primary education | ☐ Hong Kong ☐ Mainland China ☐ Elsewhere: :__________ (please specify) | |

| **From your past experience, which language(s) did you generally speak when learning science at different grades?** | | | | | |
| --- | --- | --- | --- | --- | --- |
|  | Always English | Usually English | Code-switching between English & Cantonese/Putonghua | Usually Cantonese/  Putonghua | Always Cantonese/  Putonghua |
| 1. Kindergarten | ☐ | ☐ | ☐ | ☐ | ☐ |
| 1. Primary school (Primary 1 to 6): | ☐ | ☐ | ☐ | ☐ | ☐ |
| 1. Junior secondary (Form 1) | ☐ | ☐ | ☐ | ☐ | ☐ |
| 1. Junior secondary (Form 2) | ☐ | ☐ | ☐ | ☐ | ☐ |
| 1. Junior secondary (Form 3) | ☐ | ☐ | ☐ | ☐ | ☐ |
| 1. Senior secondary (Form 4) | ☐ | ☐ | ☐ | ☐ | ☐ |
| 1. Senior secondary (Form 5) | ☐ | ☐ | ☐ | ☐ | ☐ |
| 1. Senior secondary (Form 6) | ☐ | ☐ | ☐ | ☐ | ☐ |

| How easy or difficult do you find the following speaking abilities | | | | | |
| --- | --- | --- | --- | --- | --- |
|  | Very easy | Easy | Neutral | Difficult | Very difficult |
| 1. asking questions | ☐ | ☐ | ☐ | ☐ | ☐ |
| 1. presenting a persuasive argument | ☐ | ☐ | ☐ | ☐ | ☐ |
| 1. communicating your ideas fluently | ☐ | ☐ | ☐ | ☐ | ☐ |
| 1. communicating your ideas confidently | ☐ | ☐ | ☐ | ☐ | ☐ |
| 1. presenting information to peers/classmates | ☐ | ☐ | ☐ | ☐ | ☐ |
| 1. presenting information to lab technicians | ☐ | ☐ | ☐ | ☐ | ☐ |
| 1. communicating with practitioners (e.g. scientists, patients, clients) | ☐ | ☐ | ☐ | ☐ | ☐ |

| Based on your experience in current university science classroom, please rank the following challenges related to studying science through English. | (1) NOT Challenging 🡨🡪 Most Challenging (5) | | | | |
| --- | --- | --- | --- | --- | --- |
|  | 1 | 2 | 3 | 4 | 5 |
| 1. Understanding the meaning of technical terminology in English | ☐ | ☐ | ☐ | ☐ | ☐ |
| 1. Understanding the course contents in English | ☐ | ☐ | ☐ | ☐ | ☐ |
| 1. The teaching methods/styles of an individual teacher | ☐ | ☐ | ☐ | ☐ | ☐ |
| 1. Discussions with other classmates about science in English | ☐ | ☐ | ☐ | ☐ | ☐ |
| 1. Understanding Course contents and subject-specific facts | ☐ | ☐ | ☐ | ☐ | ☐ |
| 1. Explaining topics after reading materials | ☐ | ☐ | ☐ | ☐ | ☐ |
| 1. Studying to meet teacher’s expectations in a science topic | ☐ | ☐ | ☐ | ☐ | ☐ |
| 1. Understanding exam questions in English | ☐ | ☐ | ☐ | ☐ | ☐ |
| 1. Expressing the right answers in English | ☐ | ☐ | ☐ | ☐ | ☐ |
| 1. Calculating and solving problems in English | ☐ | ☐ | ☐ | ☐ | ☐ |
| 1. Expressing my ideas through English | ☐ | ☐ | ☐ | ☐ | ☐ |
| 1. Using the correct English vocabulary and technical terms in my writing | ☐ | ☐ | ☐ | ☐ | ☐ |

| Based on your current experiences, please indicate how frequent (A) do you use the following strategies and how useful (B) they are for helping you to study science in English? | (Never (0) 🡨 🡪 Always (4)  Frequency | | | | |  |
| --- | --- | --- | --- | --- | --- | --- |
|  | 1 | 2 | 3 | 4 | 5 | |
| 1. Seeking supports from English language teachers on learning science through English | ☐ | ☐ | ☐ | ☐ | ☐ | |
| 1. Requesting additional English explanations from my science teacher | ☐ | ☐ | ☐ | ☐ | ☐ | |
| 1. Requesting additional Chinese explanations from my science teacher | ☐ | ☐ | ☐ | ☐ | ☐ | |
| 1. Attending tutorials and seek for study assistants outside school | ☐ | ☐ | ☐ | ☐ | ☐ | |
| 1. Requesting lessons materials printed in bilingual languages (Chinese and English) | ☐ | ☐ | ☐ | ☐ | ☐ | |
| 1. Asking my science teachers for feedbacks on and corrections of my science writing in English | ☐ | ☐ | ☐ | ☐ | ☐ | |
